# Preliminary screening of biomarkers in HAPE based on quasi-targeted metabolomics

**DOI:** 10.3389/fphys.2023.1122026

**Published:** 2023-03-09

**Authors:** Xue Lin, Chongyang Dai, Zhi Chen, Tongzuo Zhang, Xiaoyan Pu

**Affiliations:** ^1^ Department of Basic Medicine, Medical College of Qinghai University, Xining, Qinghai Province, China; ^2^ West China Hospital, Sichuan University, Chengdu, Sichuan Provience, China; ^3^ College of Life Science, Qinghai Normal University, Xining, Qinghai Province, China; ^4^ Northwest Institute of Plateau Biology, Chinese Academy of Sciences, Xining, Qinghai Province, China

**Keywords:** high altitude pulmonary edema, quasi-targeted metabolomics, arterial-veinal blood differences, machine algorithms, biomarkers

## Abstract

High altitude pulmonary edema (HAPE) is a serious threat to the physical and mental health of people who quickly enter high plateaus, deserves more attention and in-depth research. In our study, through the detection of various physiological indexes and other phenotypes in a HAPE rat model, the HAPE group showed a significant decrease in oxygen partial pressure and oxygen saturation, and a significant increase in pulmonary artery pressure and lung tissue water content. The lung histomorphology showed characteristics such as pulmonary interstitial thickening and inflammatory cell infiltration. We applied quasi-targeted metabolomics to compare and analyze the components of metabolites in arterial–veinous blood in control rats and HAPE rats. Using kyoto Encyclopedia of Genes Genomes (KEGG) enrichment analysis and two machine algorithms, we speculate that after hypoxic stress and comparing arterial blood and venous blood products in rats, the metabolites were richer, indicating that normal physiological activities, such as metabolism and pulmonary circulationhad a greater impact after hypoxic stress; D-mannose^DOWN^, oxidized glutathione^DOWN^, glutathione disulfide^DOWN^, and dehydrocholic acid^DOWN^ in arterial blood play key roles in predicting the occurrence of HAPE; in venous blood, L-leucine^DOWN^, L-thyroxine^DOWN^, and cis-4-hydroxy- D-proline^DOWN^ may have key roles, which can be considered biomarkers of HAPE. This result provides a new perspective for the further diagnosis and treatment of plateau disease and lays a strong foundation for further research.

## 1 Introduction

Acute mountain sickness is a serious threat to the physical and mental health of people who quickly enter high plateaus. As a severe type of acute altitude sickness, HAPE can occur in first-time visitors or in those who return to high plateaus after living at lower altitudes (Jensen and Vincent, 2022). HAPE is caused by sudden exposure to the plateau environment, which causes pulmonary artery pressure, pulmonary blood volume, pulmonary circulation disorders, and microcirculation internal fluid leakage to the lung interstitia and alveoli (Gudbjartsson et al., 2019).

As the final product of gene expression, metabolites have become the main focus in related diseases in recent years. And with the continuous development of metabolomics, more and more evidence shows that metabolites play an important role in the development of diseases. For example, untargeted metabolomics analysis of lung tumors showed that compared with normal lung tissue of mice, glutathione was increased in tumors and accumulated in NSCLC lesions (Luengo et al., 2019). The study of Schaarschmidt B et al. also suggested that targeted metabolomics are an emerging field and can be used to diagnose or assess stages and severity of different liver diseases such as cirrhosis and fibrosis (Schaarschmidt et al., 2018). Hocher B et al. reviewed the metabonomic characteristics of non-diabetes chronic kidney disease (CKD) such as IgA nephropathy, and revealing amino acids and their metabolites, tryptophan metabolites, uric acid and other purine metabolites, lipids and acylcarnitines as promising markers (Hocher and Adamski, 2017). While, there are few studies on the changes of metabolites in the process of HAPE. In 2012, Luo Y et al. (Luo et al., 2012) found the changes of some metabolites in the plasma of HAPE patients by using proton (^1^H) NMR metabolomics. However, the related components of metabolites in arterial and venous blood during HAPE are not clear. We used quasi-targeted metabolomics of metabolites in normal rats and HAPE blood to further clarify the metabolism changes and preliminarily screen for biomarkers of HAPE. Our findings could have great clinical importance in studying the pathogenesis and treatment of HAPE.

## 2 Materials and methods

### 2.1 Animals

60 specific pathogen-free (SPF) Sprague–Dawley rats, weighing 180–220g, were purchased from the *Laboratory Animal Center of Beijing Weitong Lihua Experimental Animal Technology Co., Ltd*. License No. SCXK (Beijing) 2021–0011. The feeding environment was 25°C ± 1°C, relative humidity 50%–60%, and light/darkness for 12 h circulation. Rats are allowed to eat and drink freely. Before treatment, the body quality of rats was monitored. Animals and experimental protocol were conducted according to the guidelines and ethical standards of the Animal Care and Use Ethics Committees and were approved by the Science and Technology Ethics Committee of Qinghai University.

### 2.2 Establishment and grouping of animal models

60 rats were randomly divided into two groups: the control group and the HAPE group. Control rats were kept in the animal room of the Medical College of Qinghai University. The HAPE model rats were treated in a 6,000 m, 0.6 m/s hypobaric chamber with hypoxic stress for 48 h to establish a lung injury rat model. Rat weights in each group were measured daily at a fixed point during rearing. After treatment, the rats were anesthetized *via* intraperitoneal injection of pentobarbital sodium at 45 mg/kg. The arterial blood and venous blood of the rats were collected in a heparin sodium collector, and the control group was randomly divided into a blank control venous blood group (CV group) and an arterial blood group (CA group). The HAPE group was randomly divided into a HAPE venous blood group (HV group) and a HAPE arterial blood group (HA group). Whole blood was extracted from the abdominal aorta using a blood collector, and 1 ml of blood was taken for analysis of oxygen saturation and partial pressure of oxygen in the abdominal aorta using an automatic blood gas analyzer (Sysmex, Japan). The remaining whole blood was centrifuged at 3,000 rpm for 10 min at 4°C, and the serum was collected and stored at −20°C. In addition, changes in pulmonary arterial pressure waveforms were observed using PowerLab physiological loggers (ADI, Australia), and pulmonary arterial pressure was measured using a pressure sensor. Finally, the lung tissue was collected, and the water content of the lung was calculated; the lung tissue was used for subsequent experiments.

### 2.3 Hematoxylin and eosin (H&E) staining

Lung tissue sections were dewaxed and stained with H&E staining (Servicebio, China) for pathological studies. Each lung tissue section was evaluated using a trinocular microscope (BA200Digital, Mike Audi, China).

### 2.4 Transmission electron microscope (TEM)

Lung tissue was taken from the lower tip of the right lung, cut into a 1-mm three tissue block, and fixed in a frozen tube with precooled 2.5% glutaraldehyde solution for 10 h and washed with PBS buffer. Tissue sections were fixed in osmic acid, dehydrated using gradient alcohol, embedded in pure acetone and mixed embedded liquid for 4 h, and then embedded overnight. Lead staining solution was used, and ultrastructural changes in the lung tissue were visualized using a TEM.

### 2.5 Metabolomics and LC-MS analysis

The samples (100 μL) were placed in the EP tubes and resuspended with prechilled 80% methanol by a good vortex. Then the samples were incubated on ice for 5 min and centrifuged at 15,000 g, 4°C for 20 min. Some of the supernatant was diluted to a final concentration containing 53% methanol by LC-MS grade water. The samples were subsequently transferred to a fresh Eppendorf tube and then were centrifuged at 15,000 g, 4°C for 20 min. Finally, LC-MS/MS (Want et al., 2010; Dunn et al., 2011) analyses were performed using an ExionLC™ AD system (SCIEX) coupled with a QTRAP® 6,500 + mass spectrometer (SCIEX) in Novogene Co., Ltd. (Beijing, China). The detection of the experimental samples using Multiple Reaction Monitoring (MRM) was based on a novocaine in-house database. The data files generated by HPLC-MS/MS were processed using the SCIEX OS Version 1.4 to integrate and correct the peak.

These metabolites were annotated using the KEGG database. Partial least squares discriminant analysis (PLS-DA) was performed at metaX. We applied univariate analysis (t-test) to calculate the statistical significance (*p*-value). The metabolites with VIP > 1 and *p* < 0.05 and fold change ≥ 2 or FC ≤ 0.5 (Sreekumar et al., 2009; Haspel et al., 2014; Heischmann et al., 2016) were considered to be differential metabolites. Volcano plots were used to filter metabolites of interest-based on Log2 (FC) and -log10 (*p*-value) of metabolites by ggplot2 in R language. The functions of these metabolites and metabolic pathways were studied using the KEGG database. The metabolic pathways enrichment of differential metabolites was performed, when the ratio was satisfied by x/n > y/n, metabolic pathways were considered as enrichment, when *p* < 0.05, metabolic pathways were considered statistically significant enrichment.

### 2.6 Biomarker screening

Two machine learning methods were selected to construct the prediction model. Differential biomarkers in the top 15 positions were screened by two researchers: random forest (RF) and support vector machines (SVM), which had a key role in the grouping. Then, we used receiver operating characteristic curve to screen the biomarkers for HAPE.

### 2.7 Statistical analysis

All data were analyzed using SPSS 22.0 (IBM, USA) statistical analysis software, and shown as mean ± standard deviation (SD). The one-way analysis of variance (ANOVA) and two-tailed Student’s t-test were applied to analyze the significant differences between the groups. *p* < 0.05 was significant difference.

## 3 Result

### 3.1 Physiological indexes and lung tissue morphology of HAPE rats

**FIGURE 1:**
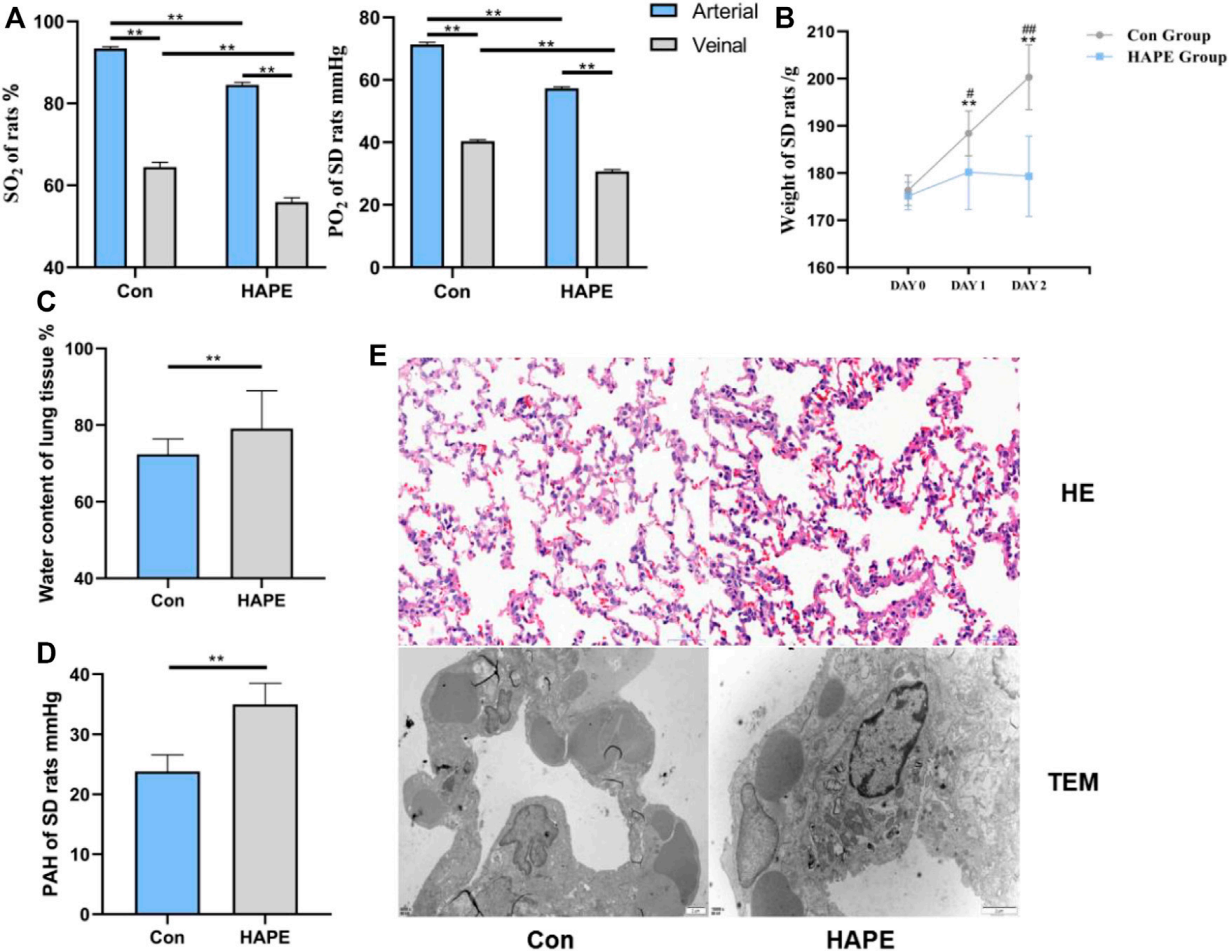
Physiological indexes and Lung tissue morphology of HAPE rats.

On analyzing blood gas in the rats, we found that the oxygen saturation and oxygen partial pressure in the venous blood (Figure 1A) of the HAPE and control groups were significantly lower than that in the arterial blood (*p* < 0.01). Through comparative analysis of the body weight of rats in each group (Figure 1B), we found that body weight in control rats increased gradually with an increasing feeding time (*p* < 0.01), whereas the body weight of rats in the HAPE group did not increase significantly with an increasing feeding time, and there was no significant difference in body weight within 3 days. However, compared with controls, the body weight of rats was significantly decreased (*p* < 0.01 or *p* < 0.05). Compared with controls, the oxygen partial pressure and oxygen saturation in the HAPE group were significantly decreased (*p* < 0.01). Compared with controls, water content of the lung tissue (Figure 1C) and the pulmonary artery pressure (Figure 1D) in HAPE rats were increased significantly (*p* < 0.01).

### 3.2 Morphological characteristics of lung tissue in HAPE rats

After H&E staining, the morphology of the rat lung tissue was observed under a light microscope, and the ultrastructural changes of the rat lung tissue were observed under a TEM. The lung tissue of the control group showed normal alveolar structure under a photoelectric microscope. Rats in the HAPE group showed injuries such as widening of the alveolar septum, infiltration of a large number of red blood cells, and inflammatory cells under light microscopy. Obvious swelling of mitochondria and shedding of lamellar bodies were observed *via* TEM (Figure 1E).

### 3.3 Analysis of the metabolome data

#### 3.3.1 QC analysis

**FIGURE 2:**
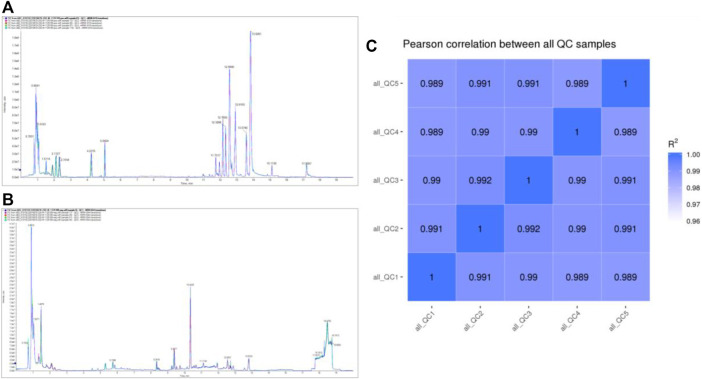
QC analysis of the metabolome data.

TIC overlapping display analysis shows that the technical repeatability of metabolites is good (Figure 2A; Figure 2B). The Pearson correlation coefficients of QC samples calculated by the relative quantitative values of metabolites are between 0.989–1.000 (Figure 2C), suggesting that the better the stability of the whole detection process, the higher the data quality. The above data indicate that the quality of this data is high, laying the foundation for follow-up relevant research.

#### 3.3.2 Basic information of differential metabolites

**FIGURE 3:**
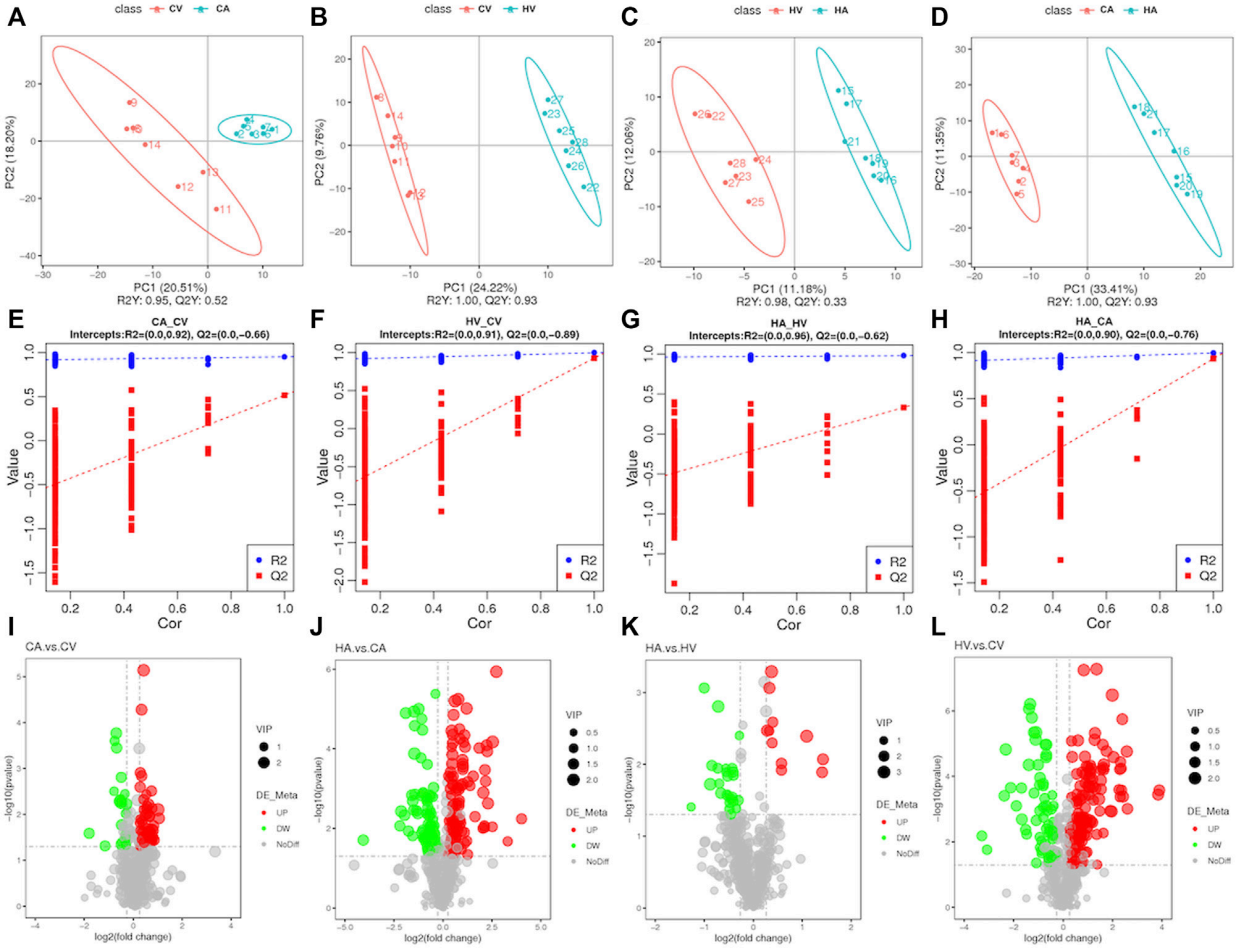
Basic information of differential metabolites.

We applied PLS-DA to each of the four groups for statistical analysis (Figures 3A–D), and obtained model evaluation parameters R2 and Q2 by 7-fold cross-validation(Figures 3E–H), with the results suggesting that the models were all over-fitted and could proceed to the next step of analysis. After perfecting the analysis of targeted metabolic data, we found that the difference in metabolites between groups was obvious, among which a total of 664 metabolites were tested (Figures 3I–3L; Supplementary Figure S1). The total difference in metabolites in CV versus CA was 74, of which 52 metabolites showed an upward trend and 22 metabolites showed a downward trend. HV versus HA totaled 36 differential metabolites, among which 11 metabolites showed an upward trend and 25 metabolites showed a downward trend. For CA versus HA, a total of 217 differential metabolites were observed, of which 155 metabolites showed an upward trend, and 62 metabolites showed a downward trend. For CV versus HV, the total difference in metabolites was 195, of which 113 metabolites showed an upward trend, and 82 metabolites showed a downward trend. In a comparison among groups, the differences of different products among groups were further clarified.

We found that differences in the composition of arterial blood and venous blood metabolites in rats after hypoxic stress tended to decline compared with that in rats in the normal group. However, after hypoxic stress and comparing arterial blood and venous blood products in rats with normal rats, the metabolites were richer, indicating that normal physiological activities, such as metabolism and pulmonary circulation after hypoxic stress, had a greater impact.

#### 3.3.3 KEGG enrichment analysis in different groups

**FIGURE 4:**
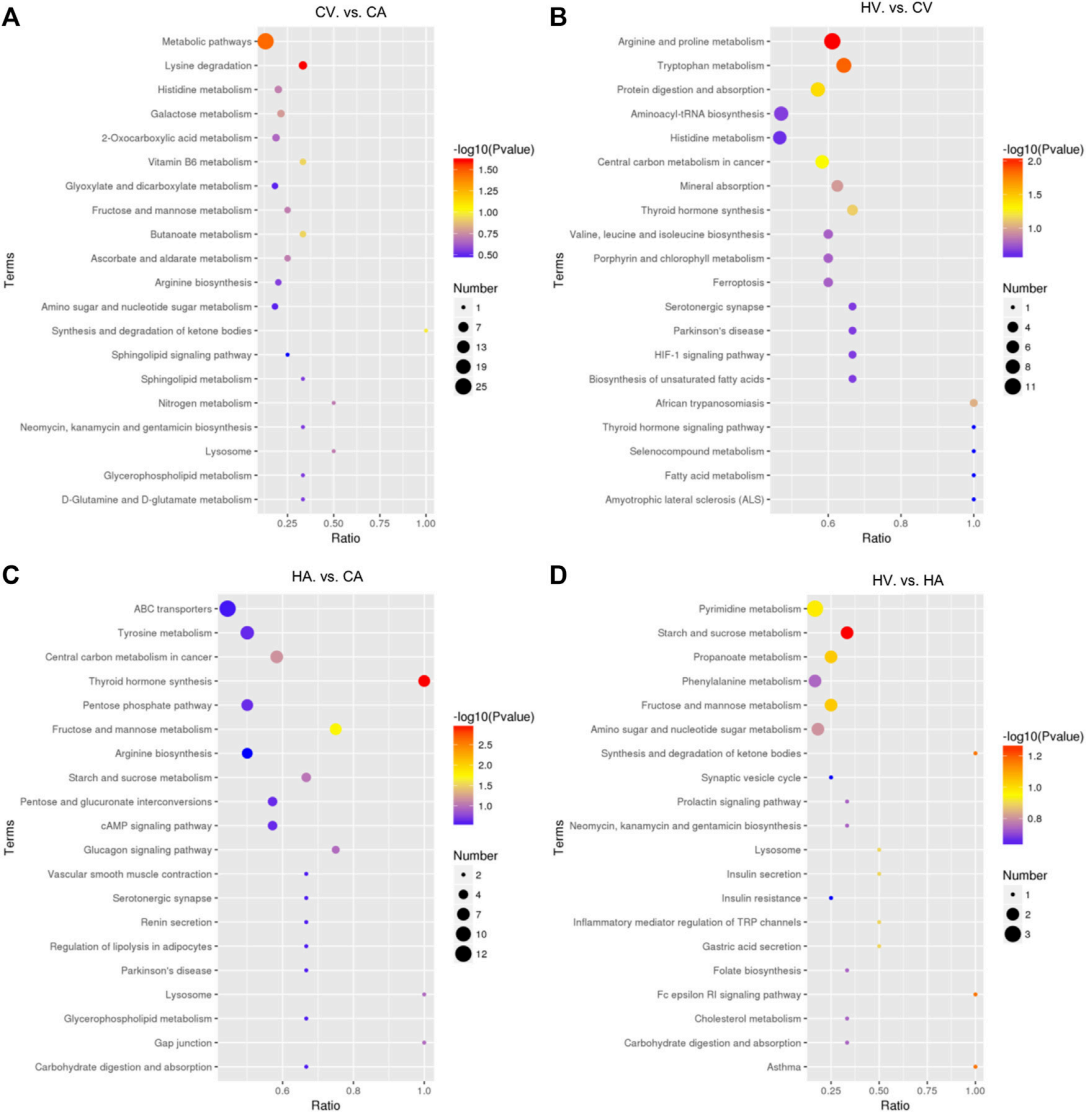
KEGG Enrichment analysis in different groups.

After KEGG enrichment analysis of differential metabolites (Figure 4), compared with the CA group, the CV group had differential metabolites that were mainly enriched in lysine (Lys) degradation and in a wide range of metabolic pathways (*p* < 0.05). The difference between the HV group and CV group was mainly enriched arginine (Arg) and proline (Pro) metabolism, tryptophan (Trp) metabolism, and the protein digestion and absorption pathway (*p* < 0.05), metabolites were also enriched and differentially expressed in central carbon metabolism and thyroid hormone (TH) synthesis in cancer (*p* > 0.05). Comparing the HA group with the CA group, differential metabolites were mainly enriched in TH synthesis and fructose and mannose metabolic pathways (*p* < 0.05), metabolites were also enriched and differentially expressed in the central carbon metabolism and in starch and sucrose metabolic pathways in cancer (*p* > 0.05). Compared with the HA group, starch and sucrose metabolism, synthesis and degradation of ketone bodies, the FC εRI signaling pathway, asthma, fructose and mannose metabolism, propanoate metabolism, and other metabolic pathways were mainly enriched in the HV group, but with *p* > 0.05, suggesting no statistical significance.

#### 3.3.4 Analysis of the key metabolites

**FIGURE 5:**
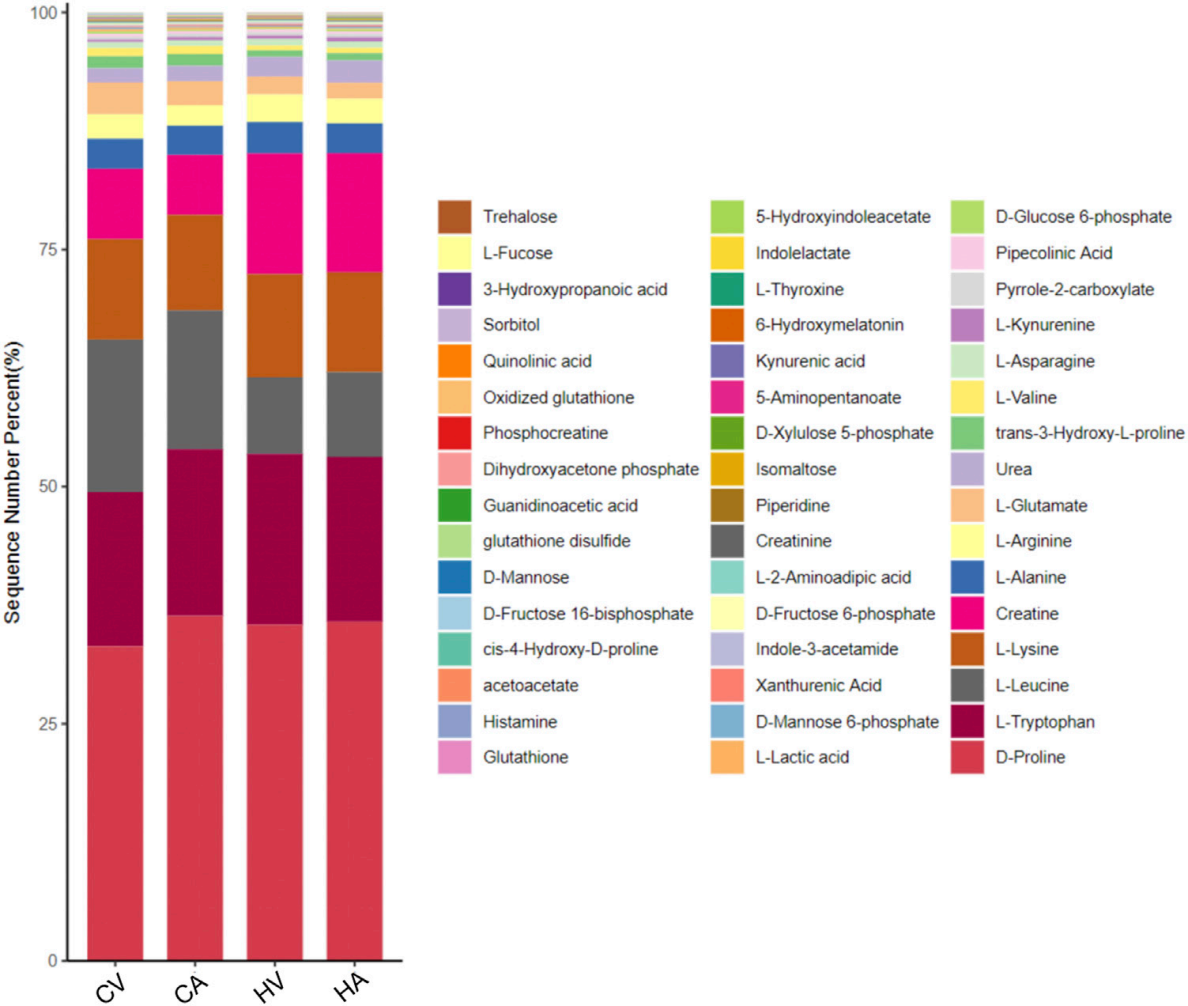
The key percentage stacking map of metabolites based on the KEGG pathways.

Key differential metabolic pathways were screened based on KEGG enrichment analysis, and the key metabolites in the pathway were screened for further analysis (Supplementary Figure S2), the percentage stacking map of metabolites is shown in Figure 5.

##### 3.3.4.1 Three major nutrients

Glucose metabolism is the core of body metabolism. Sequencing data of quasi-targeted metabolomics in the arteriovenous blood of the normal body show that the levels of glucose-related metabolites in venous blood are significantly higher than those in arterial blood, which is mainly related to micro-circulation in the body. After hypoxia stimulation, glucose metabolism obviously strengthens, such as D-glucose 6-phosphate (G6P), D-mannose 6-phosphate (M6P), D-fructose 6-phosphate (F6P), with the levels of glucose-related metabolites in both arterial blood and venous blood significantly higher than those in normal controls.

Levels of acetoacetate (ACAC) and other ketones in arterial blood were significantly higher than those in venous blood in the present study. After hypoxia stress, the level of ACAC in the arterial and venous blood of the body was significantly higher than that in the control group, suggesting that ketone metabolism in the body is further activated after hypoxia stimulation.

The KEGG enrichment analysis of the metabolome data of amino acids showed that serum differential products in the hypoxia group were mainly enriched in the metabolic pathways of Arg, Lys, Trp and revealed disorders of Arg and histidine (His) metabolism. Specifically, under hypoxia stress, the expression of key amino acids, such as citrulline (Ccp), Lys, Pro, alanine (Ala), glycine (Gly), cystine (Cys), Arg, aspartic acid (Asp), and n-acetyl-l-tyrosine (N-Ac-L-Tyr) in rat arterial serum was increased significantly, whereas the expression of lysine butyric acid, sodium glutamate (MSG), isoleucine (Ile), and acetylneuraminic acid (ANA) decreased significantly.

##### 3.3.4.2 Antioxidant substances

Compared with the control group, the concentrations of glutathione (GSH) and oxidized glutathione (GSSG) in the serum of HAPE rats were decreased significantly in both arterial blood and venous blood. In addition to GSH, lipoic acid (ALA) and other antioxidants were significantly decreased in the serum of the HAPE rat model. Interestingly, however, the concentration of vitamin C (VC), with strong antioxidant capacity, increased in rats of HAPE.

##### 3.3.4.3 Bile acid

Through analysis of quasi-targeted metabolomics data of arteriovenous blood in HAPE rats, we monitored 27 kinds of bile acids (BA). The specific results are as follows: no difference was found for BA in arterio and venous blood of normal rats; however, there were significant changes in BA metabolism components in the arteriovenous blood of HAPE rats. Among them, levels of taurocholic acid (TCA) and sodium taurodeoxycholate (TUDCA) in the arterial group were significantly higher than those in the venous group, with glycodeoxycholic acid (GDCA) concentrations decreased significantly. After hypoxia stimulation, compared with the control group, Chenodeoxycholic acid (CDCA), deoxycholic acid (DCA), 3β-ursodeoxycholic acid (3β-UDCA), and sodium taurine porcine deoxycholic acid (THDCA) showed a significant upward trend; 3α,6α,7α-trihydroxy-5β-cholic acid and beta mouse cholic acid (β-MCA) showed a significant downward trend, which suggested that significant changes take place in the BA pool during HAPE.

##### 3.3.4.4 Others

In the HAPE rat model, serum levothyroxine (L-T4) in both the HA group and HV group showed a significant downward trend compared with the control group, and the level of vitamin B2 (VB2) in rat venous blood decreased significantly in HAPE rats.

#### 3.3.5 Machine algorithms for HAPE biomarkers prediction

**FIGURE 6:**
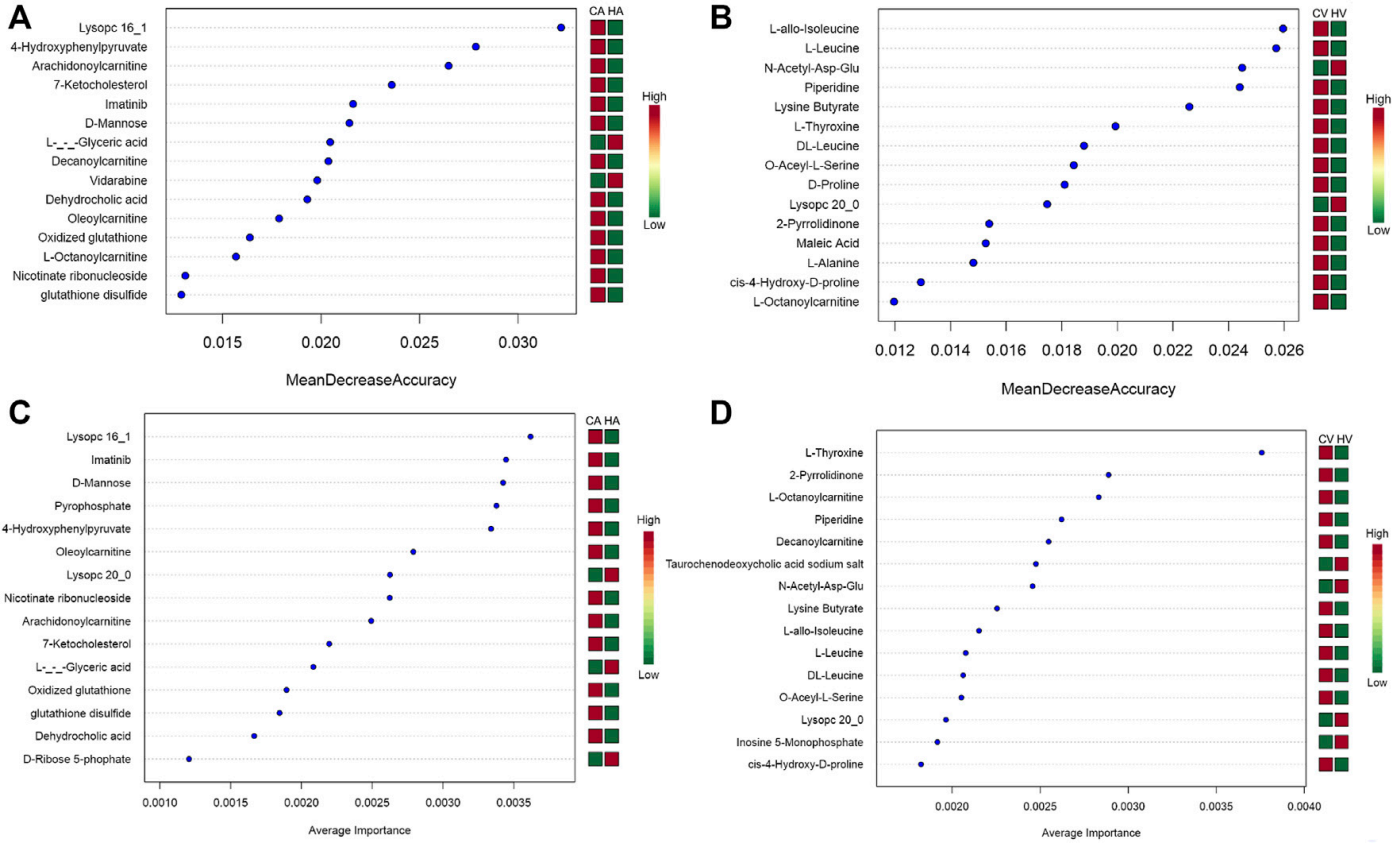
Machine Algorithms for HAPE biomarkers prediction.

The metabolites with variable importance in projection (VIP) > 1.5 and *p* < 0.01 and fold change ≥ 2 or FC ≤ 0.5 were chosen and comparisons made among each group using RF (Figures 6A,B) and SVM (Figures 6C,D) to screen the top 15 metabolites. After combined analysis of the two machine algorithms, the representative difference products between the CA and HA groups were L-(−)-glyceric acid (L-GA), imatinib (IMA), lysoPC 16:1 (Lyp 16:1), oleoylcarnitine, 4-hydroxyphenylpyruvate (4-HPPA), D-mannose, arachidonoylcarnitine, glutathione disulfide, GSSG, dehydrocholic acid (DHCA), and nicotinate ribonucleoside (NAR). The representative difference products between the CV and HV groups were piperidine (PIP), O-acetyl-L-serine (OAS), N-acetyl-asp-glu (NAAG), lysoPC 20:0 (Lyp 20:0), lysine butyrate, L-T4, L-octanoylcarnitine, L-leucine (L-Leu), L-allo-isoleucine(L-allo-Ile), DL-leucine(DL-Leu), cis-4-hydroxy-D-proline (4-D-Hyp), and 2-pyrrolidinone (2-P). We combined RF and SVM to screen out relevant metabolites and estimate AUC values (Table 1).

**TABLE 1:** ROC of the key metabolites based on RF and SVM.

Group	Metabolites	ROC (AUC)	Trend
HV vs. CV	L-allo-Isoleucine	0.92	down
	L-Leucine	0.92	down
	N-Acetyl-Asp-Glu	1.00	up
	Lysine Butyrate	0.98	down
	L-Thyroxine	0.94	down
	O-Aceyl-L-Serine	0.92	down
	Lysopc 20–0	1.00	up
	cis-4-Hydroxy-D-proline	0.94	down
	L-Octanoylcarnitine	0.98	down
HA vs. CA	Lysopc 16–1	1.00	down
	Imatinib	1.00	down
	D-Mannose	1.00	down
	4-Hydroxyphenylpyruvate	1.00	down
	Oleoylcarnitine	1.00	down
	Nicotinate ribonucleoside	0.98	down
	Arachidonoylcarnitine	1.00	down
	L-(−)-Glyceric acid	1.00	up
	7-Ketocholesterol	1.00	down
	Oxidized glutathione	0.96	down
	glutathione disulfide	0.96	down
	Dehydrocholic acid	0.98	down

## 4 Discussion

With rapid ascent to high altitudes, HAPE seriously threatens the physical and mental health of people. We placed rats in a simulated low-pressure oxygen chamber at an altitude of 6,000 m, after hypoxic stress for 48 h. The blood oxygen saturation and oxygen partial pressure of rats decreased significantly, the pulmonary artery pressure and lung tissue water content increased significantly, and the morphology of lung tissues showed a state of injury and edema, suggesting that this method can successfully result in the construction of a HAPE rat model, thereby laying a foundation for further study of this disease.

During the occurrence and development of HAPE, changes in metabolites of the body are obvious. These changes are related to the body’s adaptation to the hypoxic environment, the body’s inflammatory damage, and so on. Herein, we discuss different types of metabolites as follows.

### 4.1 Three major nutrients

The normal metabolism of three major nutrients (sugar, fat, and amino acids) of the human body is an important basis for the survival of the organism. Glucose metabolism is the core of body metabolism. In the rat control group, the glucose-related metabolites in venous blood were significantly higher than those in arterial blood, which is mainly related to microcirculation in the body. Under the condition of hypoxia, the metabolic law of the normal state of body changes indicates that more energy will be consumed to meet its metabolic needs (Webster Keith, 2003). After hypoxia stimulation, the glucose-related metabolites in both arterial blood and venous blood were significantly higher those in the normal control group, suggesting enhanced glycolytic activity in the body. Glycogen and gluconeogenesis are enhanced to maintain the stability of blood glucose, which helps to improve acute hypoxia tolerance and lung gas exchange and has a good effect on the advanced neural activities of hypoxic animals.

Among all ketones, those produced by fat metabolism play an important role in the energy homeostasis of the organism (Prins, 2008; Puchalska and Peter, 2017). AcAc, a key ketone, in the arterial blood was significantly higher than that in the venous blood in our study, which is related to normal microcirculation in the body. After hypoxic stress, the level of AcAc in the arterial and venous blood in the body was significantly higher than that in the control group, suggesting that ketone metabolism in the body is further activated after hypoxia stimulation. Previous studies have shown that ketone metabolism requires only a small amount of enzymatic reaction (Maalouf et al., 2007), which can efficiently complete productivity activities under hypoxia, improve metabolic efficiency, and reduce the production of reactive oxygen species (Gano Lindsey et al., 2014; Parra et al., 2017), suggesting that the activation of ketone metabolism is an adaptive regulation mode of the body in a hypoxic environment.

A high-altitude hypoxic environment has a certain impact on the metabolism of normal amino acids, but there are few amino acid metabolisms and specific regulation mechanisms in a hypoxic environment (Dahl Rasmus et al., 2019). When the body first enters the plateau, the metabolism of proteins is characterized by weakened synthesis and enhanced decomposition (Gibson et al., 1981). The levels of endogenous glycogenic amino acids (e.g., Gly, valine (Val), serine(Ser)) were significantly decreased after hypoxic stress, which may be related to the increase in gluconeogenesis of glycogenic amino acids caused by hypoxia (Panjwani et al., 2007); With the sensitivity of Ala aminotransferase to hypoxia, Ala metabolism is inhibited after hypoxia stress (Akshay et al., 2021), resulting in the accumulation of Ala in the body and a significant increase of Ala in the body. Tyrosine (Tyr) can improve the working ability of the body in cold and high-altitude environments and reduce symptoms in a reaction to high altitude (Cooper et al., 2018). After hypoxic stress, the levels of Tyr and its related derivatives in the serum were significantly decreased, suggesting that the body’s hypoxia tolerance decreased significantly or high-altitude injury had occurred in rats. Amino acids such as Asp are excitatory transmitters of the central nervous system. After hypoxia stimulation, metabolic disorders occur and amino acid levels increase significantly. Melatonin (MT) (Claustrat and Leston, 2015), which reduces the release of excitatory amino acids and which was significantly decreased in the body of the hypoxia group, further mediates the accumulation and neurotoxicity of excitatory amino acids and may even induce the occurrence of high-altitude cerebral edema (Ruan et al., 2000; Zhu and Xu, 2019).

It is worth noting that a large number of B vitamins can reduce disorders of amino acid metabolism caused by hypoxia and that appropriate supplementation of vitamin B (VB) may play a certain role against hypoxic injury in the body (Liu et al., 2018). Our metabolome data results showed that VB2 in rat venous blood decreased significantly in HAPE, suggesting that VB2 levels may decrease exhaustively in a hypoxic environment. This may be related to the degree of injury and prognosis.

### 4.2 Thyroid hormone

To adapt to the hypoxic environment at high altitudes, a series of changes will take place in the body, including complex changes in the endocrine system and metabolic function. Produced by the largest endocrine gland in the human body, TH has many biological effects on the body. Research shows that it has excitatory effects on almost all tissues and has a certain correlation with energy metabolism (proteins, sugars, and fats), thermoregulation, tissue differentiation, and growth and development of bodies (Maria et al., 2019). Hypoxia at high altitudes leads to changes in thyroid function and structure (Naeije, 2010); however, the results of previous studies on the changes in TH caused by hypoxia are inconsistent (Rawal et al., 1993; Savourey et al., 2004; Barnholt et al., 2006). Thyroid function has been shown to be enhanced, weakened, or even unchanged, which is related to many factors such as the exposure mode of hypoxia, altitude, duration, and availability of altitude adaptive training. Naoto Tani et al. (Tani et al., 2020a) found that an increase in thyroid-related hormones may indicate systemic hypoxia/ischemia, that is, thyroid-related hormones may be a marker of acute systemic hypoxia/ischemia. The metabolism in HAPE rats suggested that this may be an adaptive regulation method for cells to reduce the basic metabolic rate in a hypoxic environment and that the level of thyroid-stimulating hormone is closely related to the severity of hypoxia and prognosis of the disease. The lower the circulating levels of TH, the more serious the hypoxic injury (Tani et al., 2020b; Zeng et al., 2021). In conclusion, improving the examination of TH may further clarify the severity of hypoxia in patients with HAPE, suggesting that it may be used as a new biomarker for HAPE diagnosis.

### 4.3 Antioxidant substances

Under normal physiological conditions, the oxidation and antioxidant systems of the body are in dynamic balance. Previous studies have confirmed that hypoxic stress can induce oxidative stress in the body (Lin et al., 2020), in which GSH is a representative substance of antioxidants. It can directly remove reactive oxygen species (ROS) and protect cells from ROS damage under the action of GSH peroxidase. Under the stimulation of hypoxia, the levels of antioxidants such as GSH and GSSG in the body will decrease significantly, further leading to oxidative damage and inflammatory responses (Sinha et al., 2009; Chu et al., 2016). Compared with the control group, the levels of GSH and GSSG in the serum of HAPE rats were significantly decreased in both arterial blood and venous blood, suggesting that the antioxidant capacity of the body decreased. At present, GSH and GSSG are considered to be the main biomarkers after oxidative stress injury to tissues and cells (Coimbra-Costa et al., 2017). The above also further clarified the oxidative damage and inflammatory response in the process of HAPE occurrence and development.

In addition to GSH, ALA and other antioxidants were significantly decreased in the serum of HAPE model rats. Interestingly, however, VC levels, which have strong antioxidant capacity, increased in rats after hypoxia stimulation, which is inconsistent with some previous research results (Sureda et al., 2004; Wu et al., 2022). This may be related to the long half-life of VC and adaptive regulation of the body; however, further studies are needed to clarify this.

### 4.4 Bile acid

BA is a key component of the body’s normal metabolism. In recent years, many studies have shown that metabolic disorders of BA are related to a variety of disease states in the body (Jia et al., 2017), and the hypoxic environment also leads to disorders of the BA pool in the body (Ramakrishnan et al., 2014). Zhang et al. (Staley et al., 2017) found that this may be related to the change in intestinal flora caused by hypoxia. When the body is in a hypoxic environment, the composition and quantity of intestinal microorganisms in the body change significantly. Additionally, as the key material of BA transformation, intestinal microorganisms will inevitably affect the normal steady state of the BA pool. Significant changes take place in the BA pool during HAPE that further affect the activation of various receptors and metabolism of the abovementioned three major nutrients in the body (Xu, 2018). As an amphiphilic steroid molecule, metabolic disorders of BA lead to poor metabolism of fat and cholesterol (CHOL) (You et al., 2017; Jia et al., 2020). Previous studies have found that the steroid hormone synthesis pathway has a key role in acute hypoxic injury, and inflammatory factors in HAPE are significantly upregulated (Qian et al., 2018). The CHOL regulatory element protein 1c, a key molecule in the metabolism of CHOL and BA, can inhibit the over-activation of p38 MAPK/NF-KB (Chen et.al., 2004; Li et al., 2015), which has a role in inhibiting the inflammatory response in HAPE. As a target closely related to inflammatory factors, BA may provide a new direction for the prevention and treatment of altitude sickness. However, the specific reasons why all types of BA show different change characteristics in a hypoxic environment require further in-depth investigation, as do the differential changes in the BA pool; these may be related to many factors such as the hypoxic stress mode (time, altitude, speed of entry into high-altitude areas) and body physiological state.

There are obvious changes in the metabolites in the organism during the occurrence of HAPE, and the differences of metabolites in arteriovenous blood are also obvious. Combined with two machine algorithms and KEGG enrichment analysis, we speculate that D-mannose^DOWN^, GSSG^DOWN^, glutathione disulfide^DOWN^, and DHCA^DOWN^ in arterial blood play key roles in predicting the occurrence of HAPE, whereas in venous blood, L-Leu^DOWN^, L-T4^DOWN^, and 4-D-Hyp^DOWN^ play key roles in predicting the occurrence of HAPE.

## 5 Conclusion

At present, the complex pathogenesis of HAPE remains unclear. This study systematically analyzed the metabolites of HAPE model rats using class-targeted metabolomics data, further clarifying the changes in metabolism of the body during HAPE and preliminarily confirming that D-mannose^DOWN^, GSSG^DOWN^, glutathione disulfide^DOWN^, and DHCA^DOWN^ in arterial blood and L-Leu^DOWN^, L-T4^DOWN^, and 4-D-Hyp^DOWN^ in venous blood play key roles in predicting the occurrence of HAPE. Our results lay a strong foundation for further research. Interestingly, the biomarkers in arteriovenous blood after HAPE were inconsistent, which provides new ideas for clinical diagnosis and treatment and also points to the need for further research on this mechanism. Our findings may help in identifying useful molecular targets in the diagnosis and treatment of HAPE, providing a new perspective for further diagnosis and treatment of plateau disease. However, owing to the complexity of metabolomics data, not all metabolites were systematically analyzed in this study, only the key metabolites presented in this data were analyzed. In the follow-up study, we will further analyze various metabolites in the arteriovenous blood of HAPE rat model and people who rush to plateau, and further reveal their changes and mechanisms.

## Data Availability

The raw data supporting the conclusions of this article will be made available by the authors, without undue reservation.

## Supplementary material

Supplementary Figure S1 The heatmap of metabolites in each group. **(A, C)** Heat maps of the CA and HV groups compared with the CV group metabolites; **(B, D)** Heat maps of the HV and CA groups compared with the HA group metabolites

Supplementary Figure S2 The key metabolites expression of each group. Compared with CV group, CA group ***p* < 0.05; Compared with HA group, CA group ^##^ *p* < 0.05,CV group ^¥¥^ *p* < 0.05; Compared with HV group, CA group ^$$^ *p* < 0.05, CV group ^&&^ *p* < 0.05, HA group ^@@^ *p* < 0.05

## Glossary

AMS: Acute mountain sickness
HAPE: High altitude pulmonary edema
KEGG: Kyoto Encyclopedia of Genes and Genomes
CKD: chronic kidney disease
SPF: Specific pathogen-free
H&E: Hematoxylin and eosin
TEM: Transmission electron microscope
MRM: Multiple Reaction Monitoring
PLS-DA: Partial least squares discriminant analysis
RF: Random forest
SVM: Support vector machines
ANOVA: The one-way analysis of variance
Lys: Lysine
Arg: Arginine
Pro: Proline
Trp: Tryptophan
TH: Thyroid hormone
G6P: D-glucose 6-phosphate
M6P: D-mannose 6-phosphate
F6P: D-fructose 6-phosphate
ACAC: Acetoacetate
His: Histidine
Ccp: Citrulline
Ala: Alanine
Gly: Glycine
Cys: Cystine
Asp: Aspartic acid
N-Ac-L-Tyr: N-acetyl-l-tyrosine
MSG: Sodium glutamate
Ile: Isoleucine
ANA: Acetylneuraminic acid
GSH: Glutathione
GSSG: Oxidized glutathione
ALA: Lipoic acid
VC: Vitamin C
BA: Bile acid
TCA: Taurocholic acid
TUDCA: Sodium taurodeoxycholate
GDCA: Glycodeoxycholic acid
CDCA: Chenodeoxycholic acid
DCA: Deoxycholic acid
3β-UDCA: 3β-Ursodeoxycholic acid
THDCA: Sodium taurine porcine deoxycholic acid
β-MCA: Beta mouse cholic acid
L-T4: Levothyroxine
VB2: Vitamin B2
L-GA: L-(−)-Glyceric acid
IMA: Imatinib
Lyp 16:1: LysoPC 16:1
4-HPPA: 4- Hydroxyphenylpyruvate
DHCA: Dehydrocholic acid
NAR: Nicotinate ribonucleoside
PIP: Piperidine
OAS: O- acetyl-L-serine
NAAG: N- acetyl-asp-glu
Lyp 20:0: LysoPC 20:0
L-Leu: L- Leucine
L-allo-Ile: L-allo-isoleucine
DL-Leu: DL-Leucine
4-D-Hyp: Cis-4-hydroxy-D-proline
2-P: 2- Pyrrolidinone
Val: Valine
Ser: Serine
Tyr: Tyrosine
MT: Melatonin
VB: Vitamin B
ROS: Oxygen species
CHOL: Cholesterol
