# Supplementary figures and images for: Preliminary screening of biomarkers in HAPE based on quasi-targeted metabolomics
Source: Front Physiol. 2023 Mar 9;14:1122026. doi: 10.3389/fphys.2023.1122026 (PMC10034721; doi:10.3389/fphys.2023.1122026)

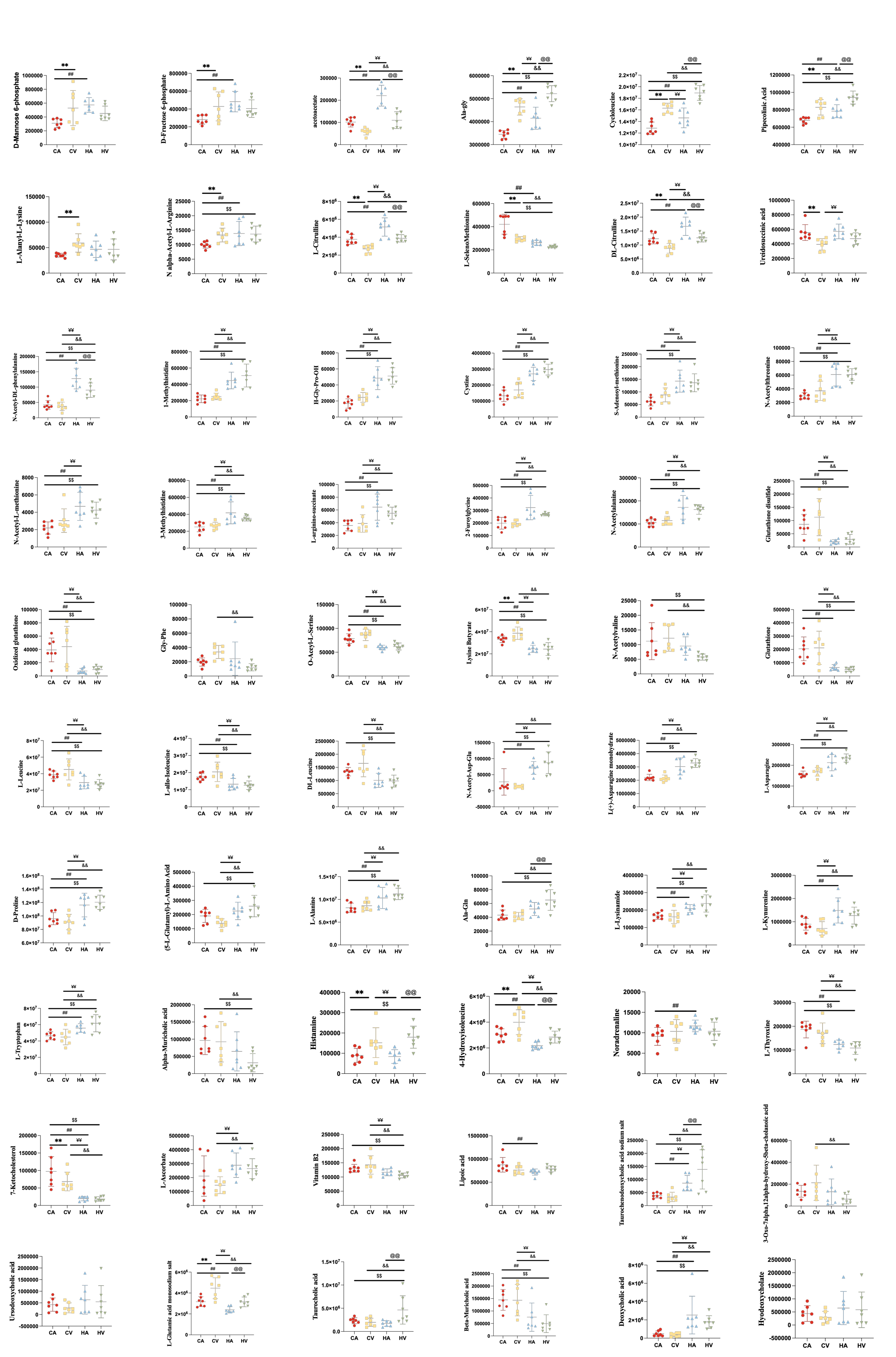

Supplement: Supplementary file 1 [file Image8.tiff]

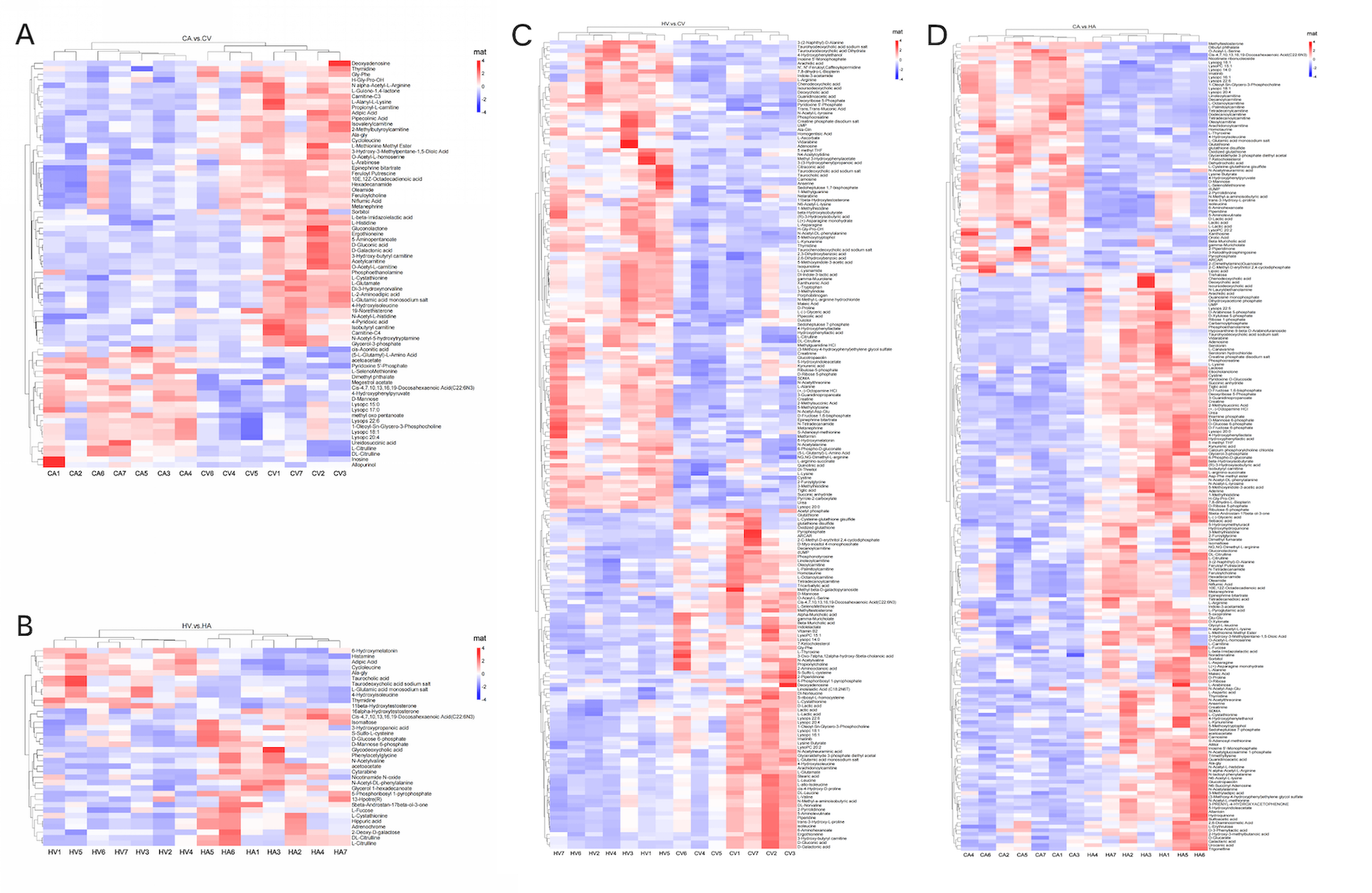

Supplement: Supplementary file 2 [file Image7.tiff]
